# Podoplanin Positive Myeloid Cells Promote Glioma Development by Immune Suppression

**DOI:** 10.3389/fonc.2019.00187

**Published:** 2019-03-26

**Authors:** Tanja Eisemann, Barbara Costa, Heike Peterziel, Peter Angel

**Affiliations:** ^1^Division of Signal Transduction and Growth Control, DKFZ/ZMBH Alliance, Heidelberg, Germany; ^2^Faculty of Biosciences, University Heidelberg, Heidelberg, Germany; ^3^Translational Program, Hopp Children's Cancer Center at NCT Heidelberg (KiTZ), University Hospital and DKFZ Heidelberg, Heidelberg, Germany; ^4^Clinical Cooperation Unit Pediatric Oncology, DKFZ, German Consortium for Translational Cancer Research (DKTK), Heidelberg, Germany

**Keywords:** tumor immunology, macrophages, immune evasion, tumor-infiltrating lymphocytes, glioblastoma, arginase, tumor microenvironment

## Abstract

The dynamic and interactive tumor microenvironment is conceived as a considerable parameter in tumor development and therapy response. Implementing this knowledge in the development of future cancer treatments could provide novel options in the combat of highly aggressive and difficult-to-treat tumors such as gliomas. One compartment of the tumor microenvironment that has gained growing interest is the immune system. As endogenous defense machinery the immune system has the capacity to fight against cancer cells. This, however, is frequently circumvented by tumor cells engaging immune-regulatory mechanisms that disable tumor-directed immune responses. Thus, in order to unlock the immune system against cancer cells, it is crucial to characterize in great detail individual tumor-associated immune cell subpopulations and dissect whether and how they influence immune evasion. In this study we investigated the function of a tumor-associated myeloid cell subpopulation characterized by podoplanin expression on the development of high-grade glioma tumors. Here, we show that the deletion of podoplanin in myeloid cells results in increased (CD8^+^) T-cell infiltrates and significantly prolonged survival in an orthotopic transplantation model. *In vitro* co-cultivation experiments indicate a podoplanin-dependent transcriptional regulation of arginase-1, a well-known player in myeloid cell-mediated immune suppression. These findings identify podoplanin positive myeloid cells as one novel mediator of the glioma-induced immune suppression. Thus, the targeted ablation of podoplanin positive myeloid cells could be included in combinatorial cancer therapies to enhance immune-mediated tumor elimination.

## Introduction

Emerging evidence supports the concept that the tumor microenvironment fundamentally impacts on tumor progression and treatment response [reviewed by (1)]. The microenvironment of most solid tumors is comprised of various non-neoplastic cell types, including fibroblasts, pericytes, endothelial, and immune cells. Reprogrammed by tumor cells, each of these stromal cell types has been shown to secrete growth factors, chemokines, anti-apoptotic, and pro-angiogenic factors or extracellular matrix components changing the local environment into a tumor-promoting milieu [as reviewed by (2–5)]. This knowledge has raised the intriguing possibility to inhibit tumor progression by targeting its supportive microenvironment. There is great hope that the development of adjuvant therapies against the tumor microenvironment will provide additional new treatment options for highly resistant and difficult to treat tumors such as gliomas.

High-grade gliomas, the most common primary brain tumors in adults, belong to the tumors with worst prognosis and highest mortality (6). Although the brain has been considered for decades as “immune privileged” with restricted access for immune cells, the immune system has more and more been recognized as a critical player in brain tumor biology (7, 8). In brain tumors, resident microglia, and infiltrated macrophages represent the most prevalent immune cells. Dependent on the genetic make-up and grade of the tumor, microglia and infiltrating macrophages can account for 30–50% of the entire tumor mass (9). Previously, it has been shown that tumor cells “educate” these myeloid cells to promote tumor cell proliferation (10), invasion (11), angiogenesis (12), and suppression of apoptosis (9). The “re-education” or depletion of tumor-associated microglia and macrophages by colony-stimulating factor 1 receptor (CSF1R) inhibition significantly reduces glioma progression (9, 13). However, there is compelling evidence that subsets of glioma-associated macrophages do not only act directly on tumor cells but furthermore create a local or even systemic immune suppression, helping tumor cells to evade rejection by the adaptive immune system (14–17). Importantly, it has been shown that microglia and macrophages do not only differ in their origin (18) but also in their response to glioma growth (17, 19), and are consequently suspected to influence tumor progression and anti-cancer therapy in different ways (20). Thus, in order to inhibit the pro-tumoral interaction of innate immune cells and tumor cells most efficiently, it is essential to comprehensively characterize individual tumor-associated immune cell subpopulations and decipher how they contribute to tumor progression.

Previously, the glioma-associated myeloid compartment has been reported to express the transmembrane protein podoplanin (PDPN) (21, 22). PDPN is a highly glycosylated protein that is constitutively expressed by various cell types of the human and rodent body. Despite its abundant expression, the function of PDPN has remained elusive for many cell types [for review see (23)]. Similarly, the effect of increased *Pdpn* expression in many pathologies has not been clarified yet. Here, *Pdpn* is expressed in neoplastic cells and cancer-associated fibroblasts of various cancer entities (24–27), in the endothelial vessel wall during venous thrombosis (28), in fibroblastic reticular cells during lymph node expansion (29) and in multiple immune cell populations (25, 30), including macrophages during inflammation (31–33). Interestingly, although PDPN on inflammatory macrophages has been reported as a critical player in the inflammation control during sepsis and acute respiratory distress syndrome (34, 35), the function of PDPN positive (PDPN^+^) macrophages in cancer has remained unexplored. Thus, in this study we examined tumor-associated PDPN^+^ myeloid cells and their effect on glioma development and immune cell infiltration. Here we show that the deletion of *Pdpn* in myeloid cells results in increased T-cell infiltrates and significantly prolonged survival, identifying the PDPN^+^ myeloid cell population as one mediator of the glioma-induced immune suppression.

## Materials and Methods

### Tumor Cell Cultivation and Transduction

*Tlx-Cre*ER^T2^; *Pten*^*flox*/*flox*^ mice (27) crossed with *Tp53*^*flox*/*flox*^ animals (The Jackson Laboratory) spontaneously developed high grade glioma tumors, from which primary murine tumor cells DKO11804 were isolated. Tumor tissue was minced and digested in Leibovitz medium supplemented with 12 U/ml papain, 100 U/ml DNase and 0.5 mM EDTA for 15 min at 37°C. After filtration (70 μm) and lysis of erythrocytes tumor cells were cultured as spheroids in DMEM/F12 medium (life technologies) containing N2 supplement (life technologies), 20 ng/ml of each EGF and FGFb (promokine), 2 mM L-glutamine and 100 U/ml penicillin/streptomycin at 37°C and 5% CO_2_. Lentiviral transduction with a construct encoding mCherry was performed in order to label the murine cells for subsequent transplantation assays. For virus production we transfected one 10 cm dish HEK293T cells with 8 μg target vector; 4 μg psPAX2; 2 μg pVSVg and 42 μg polyethylenimine (Alfa Aesar). HEK293T cells were cultivated in N2-supplemented serum-free medium. Virus-containing medium was transferred from HEK293T cells to the target cells and replaced by cultivation medium after 24 h. Upon recovery from infection recipient cells were sorted for mCherry expression by fluorescence activated cell sorting (FACS).

Established cell lines LN308; LN319; GL261 and SMA-560 were cultivated as adherent monolayers in DMEM supplemented with 10% FBS, 2 mM L-glutamine and 100 U/ml penicillin/streptomycin at 37°C and 5% CO_2_. GL261 and SMA-560 were provided by Dr. Michael Platten (DKFZ/University Hospital Heidelberg). Human glioma cell lines LN308 and LN319 were provided by Dr. Wolfgang Wick (DKFZ/University Hospital Heidelberg) and authenticated in April 2018 using Multiplex Cell Authentication by Multiplexion (Heidelberg, Germany) as described recently (36). The SNP profiles matched known profiles.

### Intracranial Injections

For orthotopic injections of DKO11804 glioma cells we used a motorized stereotaxic instrument (Neurostar). 5 × 10^5^ tumor cells were injected in 2 μl PBS 2 mm lateral (right) and 3 mm ventral to the bregma with a speed of 0.2 μl/min. Eight to ten weeks old control [*Pdpn*^*flox*/*flox*^, (37)] or conditional knockout animals (cKO, *Csf1r-Cre* (38); *Pdpn*^*flox*/*flox*^) were used as recipients. Mice were sacrificed when exhibiting termination criteria such as loss of > 20% body weight or poor general condition. Length of animal survival was measured by means of Kaplan–Meier estimate. All animal experiments were approved by the responsible authority for animal experiments (Regierungspräsidium Karlsruhe, Germany) and performed in conformity with the German Law for Animal Protection.

### Immunohistochemistry and Immunofluorescence

When animals had to be sacrificed, brains were fixed in 4% PFA and embedded in paraffin for histological examination. 6 μm histological sections were stained according to standard immunohistochemistry protocols and counterstained with haematoxylin. The antibodies used were specific for mCherry (abcam, ab167453), CD3 (Biorad, MCA1477T), Pdpn (The Developmental Studies Hybridoma Bank), and Iba1 (Wako, 01919741). The analysis of CD3^+^ cell numbers was performed using an ImageJ macro programmed by Dr. Damir Krunic (DKFZ core facility for light microscopy).

### Preparation and Flow Cytometry of Blood, Lymph Nodes, and Brain

Blood was collected in an EDTA-coated vessel and erythrocytes lysed by ACK lysing buffer. After washing in 1% FBS PBS, cells were counted.

Right mandibular lymph nodes were removed and gathered in 1% FBS PBS. Subsequently, tissue was minced between two frosted object slides which were finally rinsed with 1% FBS PBS. After filtering through a 70 μm strainer cells were once washed and counted.

Both hemispheres of unchallenged or the tumor-bearing hemisphere of treated mice were isolated and chopped using a scalpel. As the proteins of interest CD4, CD8, and PDPN were sensitive to papain treatment, we used 1.5 ml accumax supplemented with 0.75 mM EDTA and 200 U DNase I for tissue dissociation. After 15–20 min incubation at RT and thorough resuspension in 10 ml washing medium (DMEM, Sigma), cell suspension was filtered through a 70 μm cell strainer and centrifuged for 4 min at 1,500 rpm. After erythrocyte lysis and another washing step, the pellet was resuspended in 10 ml 50% isotonic Percoll/washing medium and centrifuged at 764 g at 4°C for 20 min. Half of the supernatant was removed and fresh washing medium added. After another centrifugation step of 10 min cells were counted.

Cells were stained for flow cytometry using following antibodies: CD3 (Biolegend, 100306), CD4 (eBioscience, 17-0042-81), CD8 (Biolegend, 100707), CD11b (Biolegend, 101207), CD45 (BD Biosciences, 553080), Pdpn (BioLegend, 127409), Syrian hamster isotype control (Biolegend, 402012). CD16/CD32 block (eBioscience, 14-0161-82) was performed prior to staining. Live/dead discrimination was based on 7AAD staining.

### Isolation of Microglia, Bone Marrow-Derived Macrophages, and Spleen Macrophages

Microglia cells were isolated from newborn pups (P0-P5). Brains were isolated and meninges removed. Cortices were mechanically dissociated using a glass pestle and a 70 μm cell strainer. After washing with plain DMEM medium, cell suspension of one brain was transferred in DMEM (10% FBS, 2 mM L-glutamine, 100 U/ml penicillin/streptomycin) to one poly-L-lysine coated 3 cm dish. Debris was washed off the next day and medium refreshed. By this protocol, cells are kept in co-cultivation with astrocytes (mixed glia culture).

Bone marrow-derived macrophages were generated by flushing femur and tibia of adult mice. After erythrocyte lysis and a washing step with plain DMEM, cells obtained from one leg were seeded on one non-coated 10 cm petri dish. For cultivation we used RMPI 1640 GlutaMAX (thermofisher) supplemented with 10% FBS, 100 U/ml penicillin/streptomycin, 50 mM β-mercaptoethanol and 30% L929-MCSF-conditioned medium (39).

Spleen macrophages were isolated by mechanical dissociation of adult spleens using the piston of a 5 ml syringe and a 70 μm cell strainer. After erythrocyte lysis and a washing step, cells were plated on non-coated plates. Non-adherent cells were washed off after 24 h. Spleen macrophage medium contained 10% FBS, 100 U/ml penicillin/streptomycin, 50 mM β-mercaptoethanol and 10% L929-MCSF-conditioned medium in RMPI 1640 GlutaMAX (thermofisher).

### Co-cultivation Experiments

To assess the effect of tumor cells on *Pdpn* expression of myeloid cells, 2 × 10^5^ BMDM or spleen macrophages were co-cultivated with 0.5 × 10^5^ LN308 tumor cells for 48 h in coated 6 wells. In case of microglia, LN308 were added to confluent mixed glia cultures. After 48 h, co-cultures of tumor cells and BMDM or spleen macrophages were detached by 5 min incubation with accutase and gentle usage of a cell lifter. For tumor cell and microglia co-cultures, a mild trypsinization protocol (0.05% trypsin in serum-free medium) (40) was followed in order to reduce the number of astrocytes and tumor cells in flow cytometry analysis. Subsequently, microglia cells were detached as described for the other myeloid cell types. Prior to staining of CD11b and PDPN, a CD16/CD32 block was performed.

Transwell Permeable Supports (Falcon, 353090) were utilized for indirect co-cultivation of distinct cell types to examine the effect of tumor cells on the transcription of M1/M2 markers in macrophages. 2 × 10^5^ BMDM were seeded per 6 well in above described macrophage medium. 1 × 10^5^ LN308, LN319, GL261, or SMA-560 tumor cells were seeded on top of the transwell membrane. After 72 h, the transwell and medium was removed and macrophages lysed for RNA isolation using RNeasy kit (Qiagen). Five hundred nanograms of RNA was transcribed in cDNA using RevertAid M-MuLV reverse transcriptase (Thermo Fisher Scientific).

### Quantitative Real-Time PCR

For quantitative gene expression analysis, 40 cycles of real-time PCR were performed on the StepOnePlus real-time detection system (Applied Biosystems). Every PCR reaction was carried out in duplicates with 2.5 ng of cDNA in a final volume of 12.5 μl Power SYBR® Green PCR Master Mix (Applied Biosystem). StepOneTM Software v2.2 was used for data analysis. *Ppia* was used as housekeeping gene to normalize target gene expression. We used the following primer sequences for detection of *Pdpn* transcripts (41): FW 5′-agagaacacgagagtacaacc-3′; RV 5′-caacaatgaagatccctccgac-3′, *Arg1* (42): FW 5′-TTGGGTGGATGCTCACACTG-3′; RV 5′-TTGCCCATGCAGATTCCC-3′; *Ppia*: FW 5′-AATTCATGTGCCAGGGTGGTG-3′; RV 5′-TGCCTTCTTTCACCTTCCCAA-3′. Additional primer sequences are given in Table S1.

## Results

### Glioma Tumors Harbor a PDPN^+^ Myeloid Subpopulation

**Figure 1:**
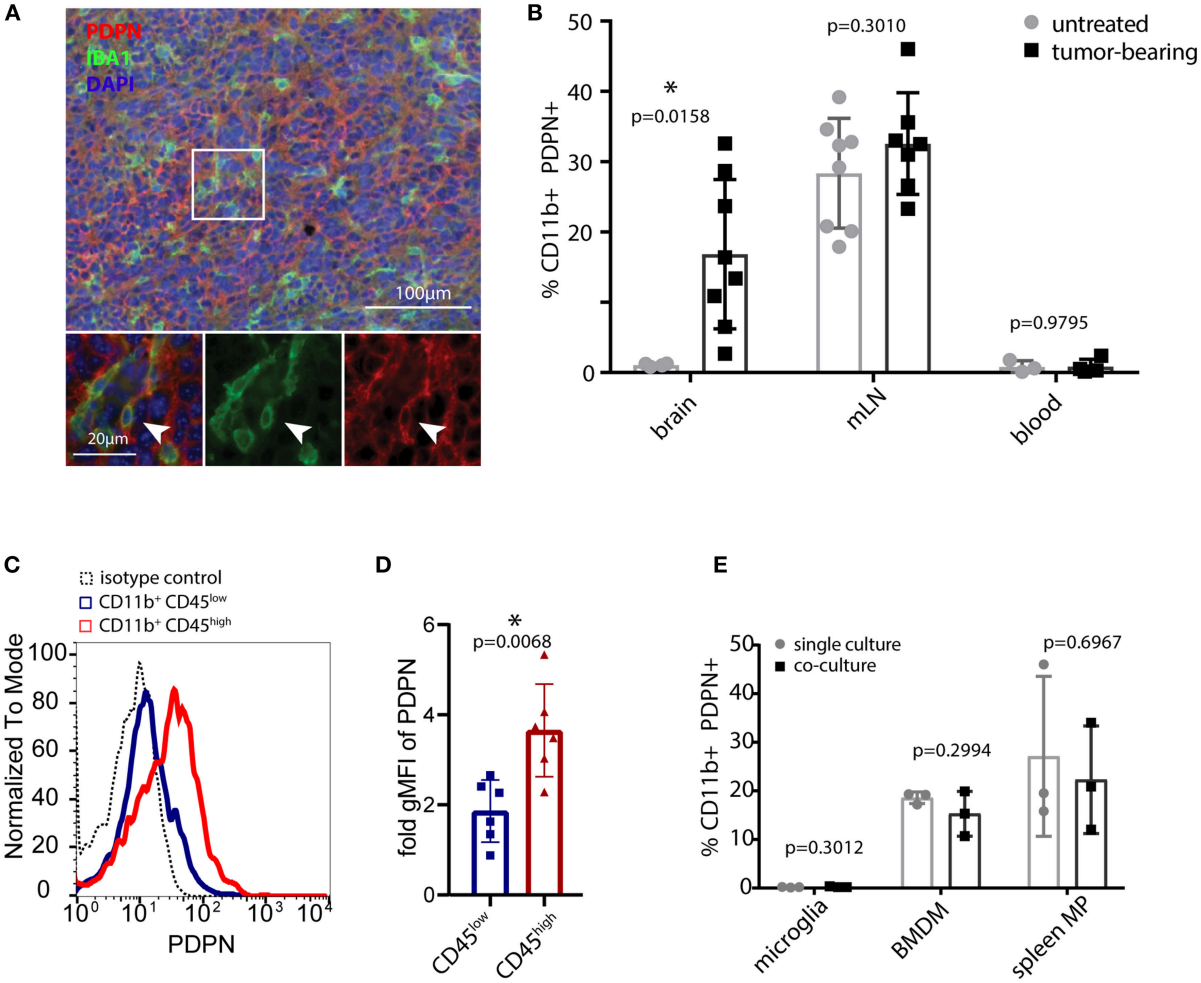
PDPN^+^ myeloid cells are present in lymphoid tissue and glioma tumors. **(A)** Immunofluorescence staining of high-grade murine glioma for PDPN (red) and IBA1 (green) shows co-expression in a subset of cells. Nuclei were stained with DAPI and pseudocolored in blue. Arrowhead indicates a cell co-expressing PDPN and IBA1. **(B)** Flow cytometry of brain, mandibular lymph nodes (mLN) and blood of untreated and tumor-bearing mice. Data represent mean and SD. Statistical analysis: Student's *t*-test. *n* = 4 and *n* = 8 for brain of untreated and tumor-bearing mice respectively. *n* = 8 and *n* = 7 mLN of untreated and tumor-bearing mice respectively. *n* = 3 and *n* = 4 for blood of untreated and tumor-bearing mice respectively. **(C)** Flow cytometry of glioma-associated myeloid cells shows PDPN is predominantly expressed by CD11b^+^ CD45^high^ cells. **(D)** Quantification of geometric mean fluorescence intensity representative for PDPN levels on CD11b^+^CD45^low^ and CD11b^+^CD45^high^ cells, normalized to isotype control staining. Statistical analysis (Student's *t*-test of logarithmized values) revealed significantly higher expression of PDPN in CD11b^+^CD45^high^ cells, *p* = 0.007; *n* = 6. **(E)** Flow cytometry of PDPN expression of microglia, bone marrow-derived macrophages (BMDM) and spleen macrophages in single- or co-culture with glioma cell line LN308. Bar graphs indicate percentage of PDPN^+^ cells of all CD11b^+^ cells, data represent mean and SD. Statistical analysis: Student's *t*-test. *n* = 3 for all samples. *p < 0.05, **p < 0.001.

We analyzed the myeloid compartment of high-grade glioma tumors obtained from orthotopic injections of syngeneic C57BL/6 glioma cells. These cells were derived from an endogenous glioma model based on the *Tlx-CreER*^*T*2^-driven deletion of *Pten* and *Tp53* in neuronal stem cells. Immunofluorescence staining (Figure 1A) and flow cytometry (Figure 1B and Figure S1A) of glioma-bearing mouse brains revealed the presence of a PDPN^+^ myeloid subpopulation. Interestingly, CD11b^+^ PDPN^+^ cells were only present in glioma-bearing brains but not in unchallenged brain tissue. This either indicates that brain tumor growth triggers *de novo Pdpn* expression in the resident microglial compartment or that the CD11b^+^ PDPN^+^ population is constituted by infiltrating myeloid cells, in line with the finding of a PDPN^+^ subpopulation of peripheral myeloid cells (in the mandibular lymph node in tumor-bearing and untreated control mice Figure 1B and Figure S1A). Of note, no PDPN^+^ myeloid cells were detected in blood (Figure 1B and Figure S1A).

To determine the PDPN^+^ myeloid cell type in gliomas we combined the CD11b and PDPN staining with CD45. Our analysis indicated that *Pdpn* is primarily expressed by CD11b^+^ CD45^high^ SSC^high^ cells (Figures 1C,D), a population that has previously been described as infiltrating macrophages (43–45). This is in line with *in vitro* experiments, where we cultivated murine microglia, bone marrow-derived macrophages (BMDM) and spleen macrophages in the absence and presence of LN308 glioma cells (Figure 1E). In contrast to BMDM and spleen macrophages that both comprise a PDPN^+^ subpopulation at any condition, we could not detect PDPN protein on microglial cells, suggesting that the PDPN^+^ myeloid cell type we found in glioma tumors are not activated microglia. Taken together, we show that gliomas harbor a myeloid subpopulation that expresses *Pdpn* and that is most likely of peripheral origin.

### PDPN^+^ Myeloid Cells Have a Tumor-Promoting Function

**Figure 2:**
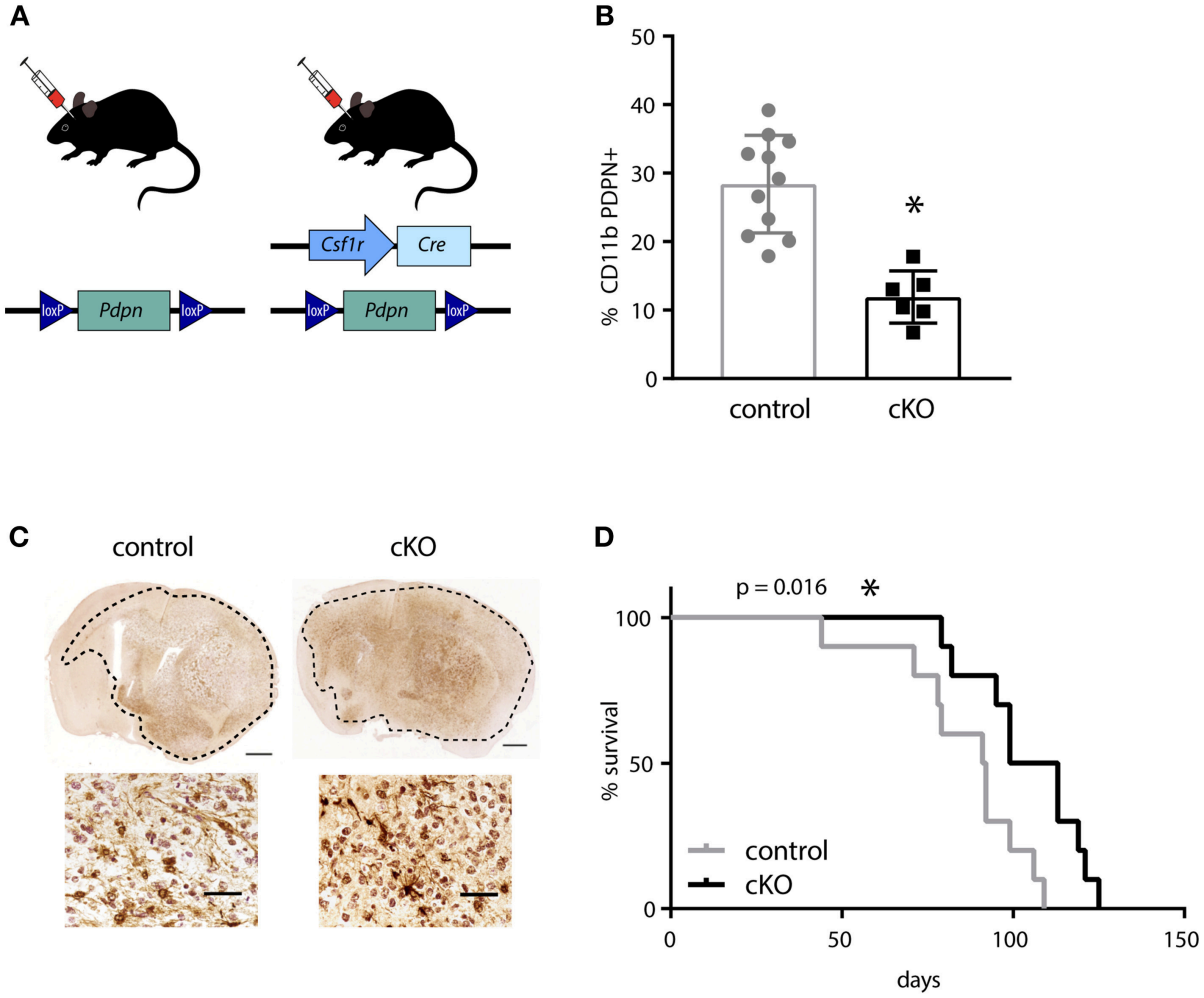
The deletion of *Pdpn* in myeloid cells improves survival of glioma bearing mice. **(A)** Schematic representation of genotypes applied for syngeneic transplantations of high-grade glioma cells. **(B)** Percentage of PDPN^+^ cells (of all CD11b^+^ cells) in mandibular lymph nodes of control and conditional knockout (cKO) animals, assessed by flow cytometry, data represent mean and SD, *p* = 0.0001; Student's *t*-test. **(C)** Immunohistochemical staining of mCherry positive glioma cells implanted in control and cKO brains, scale bar 1 mm and 50 μm for magnifications. **(D)** Kaplan-Meier survival analysis showed significantly prolonged survival of cKO animals, *p* = 0.016 (log-rank test); *n* = 10. *p < 0.05, **p < 0.001.

In order to investigate the effect of PDPN^+^ myeloid cells on glioma development, we generated a mouse line that carries a myeloid-specific deletion of *Pdpn* by crossing a *Pdpn*^*flox*/*flox*^ with a *Csf1r-Cre* transgenic line (Figure 2A). Although the colony-stimulating factor-1 receptor (CSF1R) is largely restricted to mononuclear phagocytes, the *Csf1r-Cre* line has also been reported to delete loxP-flanked alleles in lymphocytes (38). As *Pdpn* deletion in T-cells has recently been shown to significantly delay melanoma growth due to PDPN's newly discovered role as co-inhibitory receptor (46, 47), we tested whether the application of the *Csfr1-Cre* line resulted in the undesired deletion of *Pdpn* in T-lymphocytes. Flow cytometry of tumor-infiltrated CD3^+^ T-cells revealed that only a subset of ~10% expressed *Pdpn* and that this is not changed in *Csf1r-Cre; Pdpn*^*flox*/*flox*^ conditional knockout (cKO) mice (Figure S2A). In contrast, we found significantly fewer PDPN^+^ CD11b^+^ myeloid cells in mandibular lymph nodes of cKO compared to control animals (Figure 2B), albeit we could not achieve deletion of the gene in the entire population. We continued with orthotopic injections of syngeneic C57BL/6 glioma cells. These cells formed tumors with hallmarks of high-grade gliomas including extensive invasion in both control and cKO animals (Figure 2C). However, we observed a significantly prolonged survival of cKO animals (Figure 2D, *p* = 0.016). In summary, our observation of the prolonged survival of glioma-bearing cKO animals indicates that the PDPN^+^ myeloid subpopulation promotes glioma progression.

### The Deletion of *Pdpn* in Myeloid Cells Increases T-Cell Infiltrates

We next examined how the beneficial effect of the myeloid cell-specific *Pdpn* knockout is mediated. Myeloid cells, and especially macrophages, have been shown to acquire a tumor-promoting phenotype in cancer patients and mouse models of cancer (4, 20). Tumor-associated macrophages support tumor growth by diverse mechanisms of immune suppression (15, 48–50). Although there seems to be no consensus about the significance of immune infiltrates in brain tumors due to local immune suppression, there is increasing evidence that associates a beneficial outcome with increased CD3^+^/CD8^+^ infiltrates in glioblastoma patients (51, 52). Thus, we investigated T-cell numbers in control and cKO animals.

**Figure 3:**
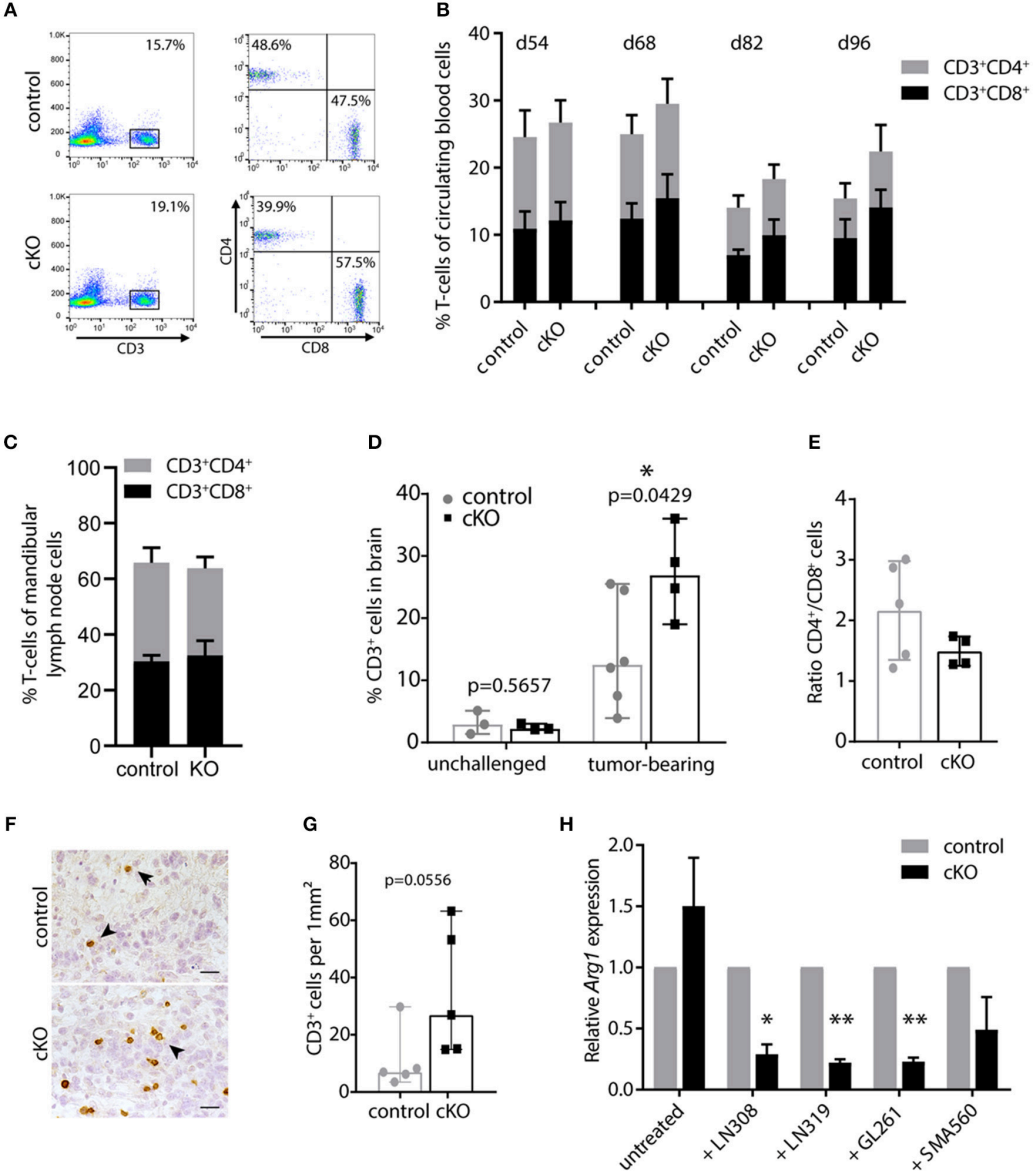
PDPN^+^ myeloid cells execute an immune inhibitory function. **(A)** Flow cytometry plots of one control and one cKO animal 82 days after tumor cell transplantation. CD3^+^ T-cells were pre-gated for lack of 7AAD and analyzed for CD4 and CD8 expression. **(B)** Time course of flow cytometric analysis of circulating CD3^+^CD4^+^ and CD3^+^CD8^+^ T-cells of tumor-bearing control and cKO animals. Days post tumor cell injection are indicated on top. Statistical comparison (Student's *t*-test) of T-cell percentages between control and cKO animals showed following *p*-values: 0.05; 0.25; 0.047 and 0.056 at d54; d68; d82 and d96, respectively, *n* = 8. **(C)** Percentage of CD3^+^CD4^+^ and CD3^+^CD8^+^ T-cells in mandibular lymph nodes is not altered between glioma-bearing control and cKO mice. Statistical comparison (Student's *t*-test) of T-cell percentages between control and cKO animals showed following *p*-value 0.6647; *n* = 5 (control) and *n* = 4 (cKO). Data **(B,C)** represent mean and SD. **(D)** Percentages of brain-infiltrating CD3^+^ T-cells are increased by tumor growth and are significantly elevated in tumors of cKO mice. Statistical analysis: Student's *t*-test, *n* = 3 (each unchallenged control and cKO); *n* = 6 (tumor-bearing control); *n* = 4 (tumor-bearing cKO). **(E)** CD4^+^/CD8^+^ ratio of CD3^+^ cells detected in the brain by flow cytometry. Statistical analysis: Student's *t*-test. *p* = 0.1609; *n* = 5 (control) and *n* = 4 (cKO). **(F)** Representative immunohistochemical staining of CD3, scale bar 25 μm **(G)** Corresponding quantification of CD3^+^ cells in tumors of control and cKO animals shows a clear tendency to increased T-cell infiltration in cKO mice (*p* = 0.0556, Mann-Whitney test, *n* = 5). Data **(D,E,G)** represent median and 95% CI. **(H)** Relative *Arg1* expression in BMDM isolated from control or cKO animals analyzed by quantitative Real-Time PCR, performed with biological triplicates. Values normalized to house-keeping gene *Ppia. Arg1* expression of cKO macrophages was normalized to expression of control macrophages for each condition. *Arg1* expression is significantly attenuated in *Pdpn* knockout macrophages when co-cultivated with LN308 (*p* = 0.0173), LN319 (*p* = 0.002), or GL261 (*p* = 0.0029), *p*-value for SMA-560-co-cultivation = 0.111, paired Student's *t*-test of logarithmized values, *n* = 3. *p < 0.05, **p < 0.001.

First, we monitored CD3^+^, CD4^+^, and CD8^+^ cells in the blood during tumor development by flow cytometry. T-cell percentages and the balance between CD4^+^ and CD8^+^ T-cells up to day 54 post tumor cell injection were comparable to unchallenged mice (data not shown). However, in subsequent analyses we found slightly decreasing T-cell numbers during tumor development in all animals, especially of the CD4^+^ compartment (Figure 3B). This is in line with peripheral lymphocyte deficiencies and CD4 lymphopenia observed in glioblastoma patients (53), indicating a systemic immune suppression. Interestingly, cKO mice showed in general a slightly higher CD3^+^ T-cell number compared to control animals (Figures 3A,B). This becomes more apparent during later stages of tumor development (*p* = 0.04 for d82 and *p* = 0.055 for d96 post tumor cell injection). Furthermore, we analyzed T-cell populations in the mandibular lymph node which has been shown to be one of the draining lymph nodes of the brain (54). When animals had to be sacrificed, the mandibular lymph nodes were removed and cells isolated for analysis by flow cytometry. Lymph nodes of the majority of analyzed cKO animals appeared to be enlarged compared to control mice (Figure S2B), indicating a response to tumor growth. However, we did not find a significant difference in the percentage of CD3^+^ T-cells or the ratio of CD4^+^/CD8^+^ cells in the lymph node when analyzed by flow cytometry (Figure 3C). Taken together, brain tumor growth seemed to impact on peripheral T-cells and PDPN^+^ myeloid cells. Control animals appeared slightly more susceptible to glioma-induced systemic immune suppression than mice carrying a myeloid-specific deletion of *Pdpn*.

We then continued analyzing the T-cell infiltration of brain tumors. Brain tissue of untreated mice contained only few CD3^+^ T-cells (~3% of isolated cells, Figure 3D). These cell numbers were elevated in the tumor setting, however, probably due to the high immune suppressive potential of glioma tumors, this increase was rather mild in tumors of control mice with a median of about 13% of all isolated cells. Remarkably, cKO animals showed significantly elevated T-cell infiltrates with about 27% CD3^+^ cells (Figure 3D). This increase in CD3^+^ immune cell infiltrates in tumors of cKO compared to control animals was also shown by immunohistochemistry staining (Figures 3F,G). Moreover, we observed that most tumors of cKO animals harbored higher numbers of infiltrated CD8^+^ T-cells, also in relation to CD4^+^ cells (lower CD4^+^/CD8^+^ ratio), than control tumors (Figure 3E). The overall increase in infiltrated T-cells, together with elevated cytotoxic CD8^+^ cell infiltrates and an altered CD4^+^/CD8^+^ ratio potentially accounts for the prolonged survival of cKO animals.

We next tried to understand how PDPN^+^ myeloid cells inhibit T-cells. Thus, we analyzed the expression of a panel of common M1 (IL-1b, IL-12, iNOS, TNFα) and M2 (TGFβ, IL-10, ARG1) phenotype markers and the immune suppressive PD-L1 (CD274) in control and *Pdpn*-deleted BMDM co-cultivated with four different glioma cell lines (Figures S2C-K). One examined marker was found to be differentially regulated between control and knockout macrophages; arginase-1 (Figure 3H and Figure S2J). We observed that PDPN-deficient macrophages show a significantly impaired transcriptional upregulation of *Arg1* expression in response to three different glioma cell lines. Tumor-associated macrophages and myeloid-derived suppressor cells have been reported to inhibit T-cell activity and viability by the secretion of ARG1 and consequent depletion of arginine (48, 55). Thus, PDPN^+^ myeloid cells might mediate their immune suppressive function by increased expression of *Arg1*, which is attenuated upon *Pdpn* deletion.

Taken together, during tumor development, we observe signs for a systemic immune suppression in both control and cKO animals, which, however, seems to be less pronounced in cKO mice. Strikingly, tumors of mice carrying a myeloid-specific deletion of *Pdpn* show higher brain tumor infiltrates of CD3^+^ and CD3^+^ CD8^+^ cells than control tumors. Moreover, an altered ratio between CD8^+^ and CD4^+^ T-cells was detected between the two groups. These findings indicate an immune suppressive function for PDPN^+^ myeloid cells. *In vitro* gene expression analysis suggests that this suppression might be mediated via increased *Arg1* expression in PDPN proficient macrophages.

## Discussion

The well-established impact of the tumor microenvironment and particularly of cells of the immune system on tumor development and therapy response facilitates the exploration of novel therapeutic approaches calling for the identification and detailed characterization of the involved cell types. Thus, in this study we investigated a myeloid subpopulation, characterized by *Pdpn* expression, that has previously been reported to critically regulate the inflammatory immune response (34, 35). As PDPN has furthermore been identified as an inhibitory surface protein on T-cells in a tumor setting (47), we questioned whether PDPN might have an immune regulatory function in tumor-associated myeloid cells as well. We performed orthotopic injections of syngeneic high-grade glioma cells, an entity that is characterized by strong local and systemic immune suppression. Here, we detected PDPN^+^ myeloid cells in glioma tumors, however, not in untreated brains. Furthermore, peripheral lymphoid tissues harbored a PDPN^+^ myeloid subpopulation in presence but also in absence of glioma growth. Together with the fact that we did not detect PDPN protein on circulating myeloid cells of the blood, we presumed that *Pdpn* expression is initiated in a subset of peripheral myeloid cells upon tissue infiltration and differentiation. This was further supported by the fact that in brain tumors, *Pdpn* is predominantly expressed by CD11b^+^ CD45^high^ SSC^high^ cells, a population that has been considered as infiltrating macrophages. However, CD45^high^ or CD45^low^ may not clearly discriminate the different myeloid subpopulations as it has been shown that tumor-associated microglia are able to upregulate CD45 expression (56). To assess whether association with tumor cells induces expression of *Pdpn*, we isolated murine microglia and two other macrophage populations, BMDM and spleen macrophages, and co-cultivated them with the glioma cell line LN308. Our result that, in contrast to macrophages, microglia did not show *Pdpn* expression at any condition suggests that PDPN^+^ myeloid cells found in brain tumor tissue are most likely of peripheral origin and recruited by tumor growth.

In our murine glioma model deletion of *Pdpn* in myeloid cells has a significantly beneficial effect on survival, suggesting a pro-tumoral role for PDPN^+^ myeloid cells. In fact, the effect on tumor growth in our genetic model may even be underestimated due to the incomplete deletion of *Pdpn*. Incomplete recombination of *Pdpn*^*flox*^ alleles in macrophages has also been reported for the combination with another transgenic *Cre*-line *Vav-Cre*; (57), making it questionable whether the employment of another *Cre*-line would have resulted in more efficient PDPN deletion.

Since GBM-infiltrating macrophages, microglia, and myeloid-derived suppressor cells have been shown to hinder T-cell proliferation, infiltration and activation (14, 16, 58) we addressed the question whether PDPN^+^ myeloid cells promote tumor development by intervention with the T-cell compartment. Therefore, we analyzed T-cell numbers of the periphery and local brain tumor tissue. In line with published data, we found decreasing T-cell percentages in blood during tumor progression, indicating a systemic immune suppression (53). Of note, most cKO animals showed a less pronounced reduction of circulating T-cells as well as enlarged mandibular lymph nodes, suggesting a general role of PDPN in the regulation of immune suppression. As we exclusively detected a difference in T-cell percentages in (late stage) tumor-bearing and not in unchallenged mice (data not shown), we presume that the PDPN^+^ myeloid cells are not involved in T-cell development per se, but interfere with T-cell activation/proliferation or survival in a tumor setting.

Consistently, the analysis of brain tumors revealed that CD3^+^ and CD8^+^ cells were more abundant in tumors of cKO mice than in controls. Of note, increased CD3^+^ and CD8^+^ infiltrates have also been associated with better prognosis of glioma patients (51, 52). Furthermore, we observed a reduced CD4^+^/CD8^+^ ratio of tumor infiltrating lymphocytes in the absence of PDPN^+^ myeloid cells. In line with this, a previous report has indicated that not only the number of T-cells but also their balance determines effective anti-tumor immunity, in particular high CD4^+^/CD8^+^ ratios contribute to poor prognosis of glioma patients (59). These findings indicate that PDPN^+^ myeloid cells contribute to immune suppression and presumably to consequent immune evasion, enhanced tumor growth and poor survival. *In vitro* characterization of PDPN^+^ and *Pdpn* knockout macrophages showed PDPN-dependent enhanced *Arg1* expression, an enzyme with known pro-tumorigenic function in the tumor microenvironment (48, 60, 61), in response to the presence of glioma cells. Consistently, Arg1 could be one mediator of the here proposed immune suppressive function of PDPN^+^ myeloid cells. Additional experiments would be required to validate this cascade *in vivo* and furthermore, to show that in the absence of PDPN^+^ myeloid cells, T-cells not only infiltrate the tumors but also execute cytotoxic reactivity against the tumor cells causing the observed prolonged survival. However, as there is increasing evidence for a beneficial outcome in glioblastoma patients with increased CD3^+^ and CD8^+^ infiltrates (51, 52), we consider it most likely that the increased infiltration of T-cells in tumors of cKO animals accounts for their prolonged survival. Furthermore, it remains to be determined whether PDPN^+^ myeloid cells need to be located within the brain to execute their proposed immune inhibitory function or whether they are able to induce a systemic immune suppression sufficient to prevent local T-cell infiltration.

Taken together, we describe a population of immune-inhibitory tumor-associated PDPN^+^ myeloid cells, which contributes to the poor prognosis in high-grade glioma. Exploiting this knowledge, PDPN^+^ myeloid cells could be targeted in combinatorial immunotherapies to enhance immune-mediated tumor destruction.

## Data Availability

All datasets generated for this study are included in the manuscript and/or the Supplementary Files.

## Author Contributions

TE, HP, and PA designed the study. TE performed experiments. BC developed the syngeneic injection model. TE, BC, HP, and PA analyzed and interpreted the data and wrote the manuscript. All authors were involved in revising the manuscript, have read and approved the final version.

### Conflict of Interest Statement

The authors declare that the research was conducted in the absence of any commercial or financial relationships that could be construed as a potential conflict of interest.
