# Supplementary material for: Podoplanin Positive Myeloid Cells Promote Glioma Development by Immune Suppression
Source: Front Oncol. 2019 Mar 26;9:187. doi: 10.3389/fonc.2019.00187 (PMC6443903; doi:10.3389/fonc.2019.00187)
Supplement: Supplementary file 1 [file Data_Sheet_1.docx]

Supplementary Material

Podoplanin positive myeloid cells promote glioma development by immune suppression

**Tanja Eisemann^1,2^, Barbara Costa^1^, Heike Peterziel^1;3;#^ , Peter Angel^1;*.#^**

*** Correspondence:**Corresponding Author
p.angel@dkfz.de

Table ST1. List of Primers used for qualitative Real-Time PCR.

| **Transcript** | **FW sequence 5‘-3‘** | **RV sequence 5‘-3** | **Reference** |
| --- | --- | --- | --- |
| *CD274* (PD-L1) | GACCAGCTTTTGAAGGGAAATG | CTGGTTGATTTTGCGGTATGG | (Barsoum et al., 2014) |
| *Nos2* | CCCTCCTGATCTTGTGTTGGA | CCACCCGAGCTCCTGGAAC |  |
| *Il-1b* | TGCCACCTTTTGACAGTGATG | AAGGTCCACGGGAAAGACAC |  |
| *Il-12* | AGTGACATGTGGAATGGCGT | GGCGGGTCTGGTTTGATGAT |  |
| *Il-10* | TAAGGGTTACTTGGGTTGCCA | CTCAGCCGCATCCTGAGG | (Textor et al., 2007) |
| *Tgfb* | GAACCAAGGAGACGGAATACAG | CAGACAGAAGTTGGCATGGTAG |  |
| *Tnfa* | AGGCACTCCCCCAAAAGATG | GCTCCTCCACTTGGTGGTTT |  |

**
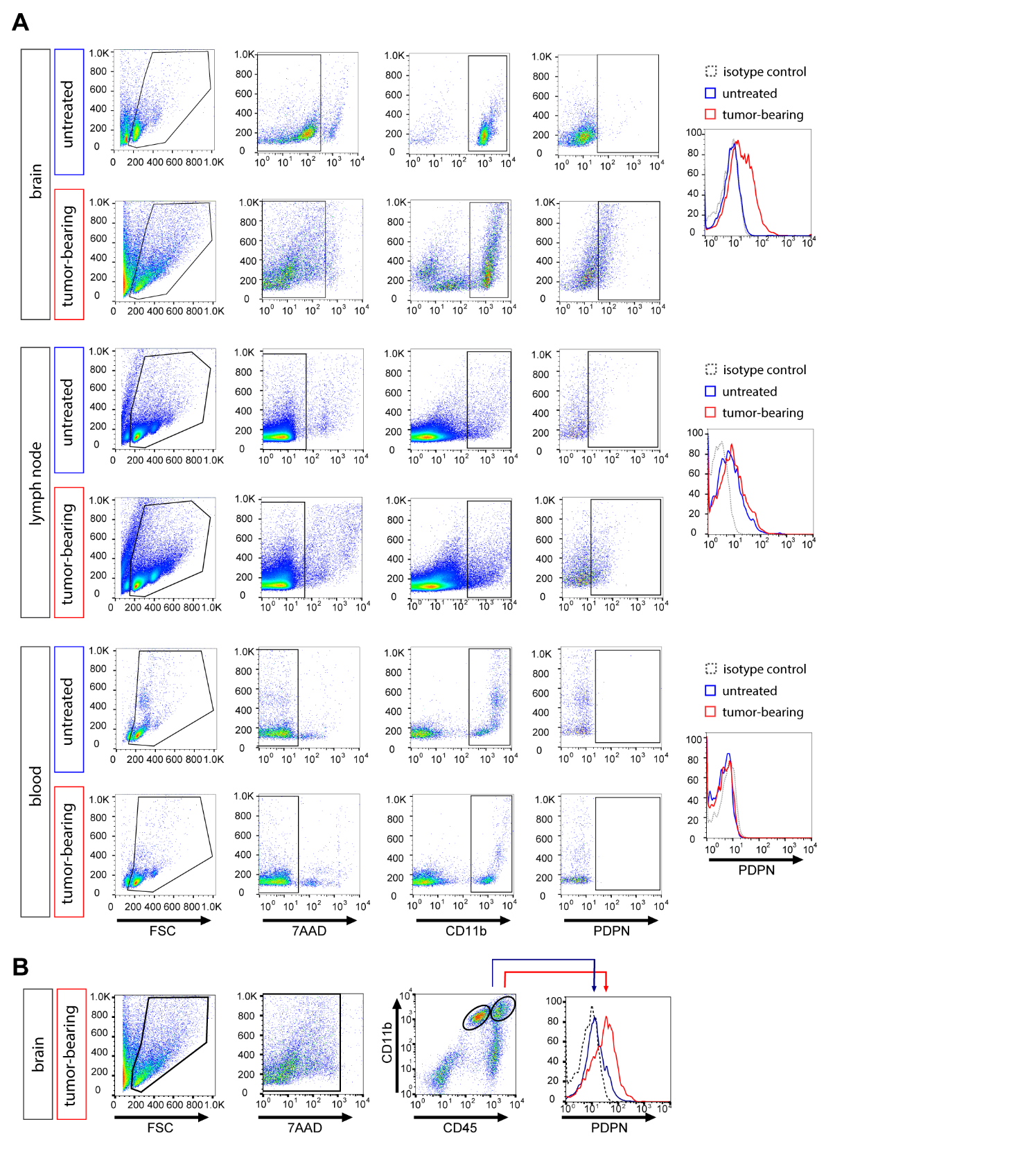
**

**Supplementary Figure 1.** (A) Flow cytometry of brain, mandibular lymph node and blood of unchallenged (blue line in histogram) and tumor-bearing mice (red). Cells were gated for lack of 7AAD (exclusion of dead cells) and CD11b expression. CD11b^+^ cells were analyzed for PDPN expression. (B) Flow cytometry of cells isolated from brain tumor. 7AAD negative cells were gated for CD11b pression and either CD45^low^ (blue line in histogram) or CD45^high^ (red) levels. These populations were subsequently analyzed for PDPN expression

**
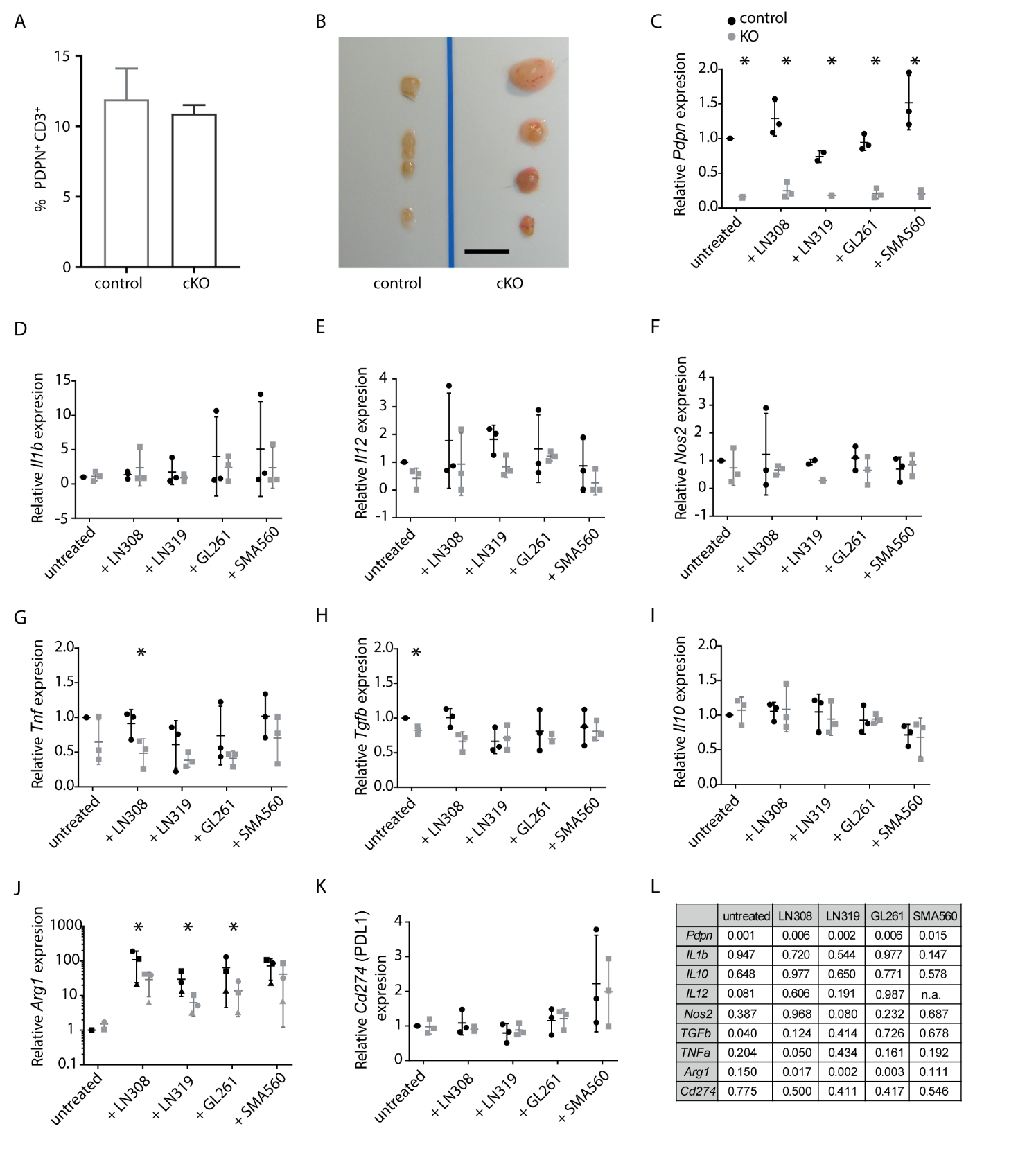
**

**Supplementary Figure 2.** (A) Flow cytometry of CD3^+^ T-cells infiltrated in tumors of control or cKO mice. Percentage of PDPN^+^ cells of all CD3^+^ cells is given, data represent mean and SD, n = 2. (B) Mandibular lymph nodes of glioma-bearing control and cKO mice, scale bar 5mm. (C) Relative *Pdpn*, (D) *Il1b*, (E) *Il12*, (F) *Nos2*, (G) *Tnfa*, (H) *Tgfb*, (I) *Il10*, (J) *Arg1*, (K) *Cd274* expression in BMDM isolated from control or cKO animals analyzed by quantitative Real-Time PCR, performed with biological triplicates. Values normalized to house-keeping gene *Ppia.* Note logarithmic axis in (J), data represent median and SD. Statistical analysis: Student’s t-test of logarithmized values, p values are given in (L).

**References**

Barsoum, I.B., Smallwood, C.A., Siemens, D.R., and Graham, C.H. (2014). A mechanism of hypoxia-mediated escape from adaptive immunity in cancer cells. *Cancer Res* 74**,** 665-674.

Textor, B., Licht, A.H., Tuckermann, J.P., Jessberger, R., Razin, E., Angel, P., Schorpp-Kistner, M., and Hartenstein, B. (2007). JunB is required for IgE-mediated degranulation and cytokine release of mast cells. *J Immunol* 179**,** 6873-6880.
